# Mitochondrial Adaptation in Skeletal Muscle: Impact of Obesity, Caloric Restriction, and Dietary Compounds

**DOI:** 10.1007/s13668-024-00555-7

**Published:** 2024-07-08

**Authors:** Lauren Jun, Ya-Xiong Tao, Thangiah Geetha, Jeganathan Ramesh Babu

**Affiliations:** 1 Department of Nutritional Sciences, Auburn University, Auburn, AL 36849 USA; 2 Department of Anatomy Physiology and Pharmacology, Auburn University, Auburn, AL 36849 USA; 3 Boshell Metabolic Diseases and Diabetes Program, Auburn University, Auburn, AL 36849 USA

**Keywords:** Mitochondrial biogenesis, Mitochondrial dynamics, Skeletal muscle fiber type, Metabolic disease, Resveratrol, Omega-3 fatty acids

## Abstract

**Purpose of Review:**

The global obesity epidemic has become a major public health concern, necessitating comprehensive research into its adverse effects on various tissues within the human body. Among these tissues, skeletal muscle has gained attention due to its susceptibility to obesity-related alterations. Mitochondria are primary source of energy production in the skeletal muscle. Healthy skeletal muscle maintains constant mitochondrial content through continuous cycle of synthesis and degradation. However, obesity has been shown to disrupt this intricate balance. This review summarizes recent findings on the impact of obesity on skeletal muscle mitochondria structure and function. In addition, we summarize the molecular mechanism of mitochondrial quality control systems and how obesity impacts these systems.

**Recent Findings:**

Recent findings show various interventions aimed at mitigating mitochondrial dysfunction in obese model, encompassing strategies including caloric restriction and various dietary compounds.

**Summary:**

Obesity has deleterious effect on skeletal muscle mitochondria by disrupting mitochondrial biogenesis and dynamics. Caloric restriction, omega-3 fatty acids, resveratrol, and other dietary compounds enhance mitochondrial function and present promising therapeutic opportunities.

## Introduction

In the era of convenience and abundance of ultra-processed foods, the Western diet has become embedded in our current society. Despite its popularity, the Western diet has been recognized as the major culprit of a more pressing matter – the global obesity epidemic. According to the World Health Organization (WHO), the global obesity rate has almost tripled from 1975 to 2016 [1], posing a significant public health concern and underscoring the need for comprehensive understanding of its multifaceted impact on our health.

Obesity is a chronic disease, defined as excessive fat accumulation [1]. At the population level, body mass index (BMI), defined as weight in kilograms divided by height in meters squared (kg/m^2^), serves as the most useful metric for assessing obesity. For adults, a BMI greater than or equal to 30 kg/m^2^ is defined as obese. Notably, obesity is frequently associated with other metabolic complications including type 2 diabetes mellitus (T2DM), non-alcoholic fatty liver disease, cardiovascular diseases, chronic kidney disease, Alzheimer’s diseases, and cancers [2]. Beyond these systemic effects, obesity also exerts deleterious consequences on specific tissues, including the skeletal muscle.

Skeletal muscle plays a crucial role in our overall health. Not only is it the largest organ in the body for locomotion, but it is also the major site for energy metabolism and hormone secretion. Skeletal muscles are composed of bundle of muscle fibers, and underneath the fibers lie mitochondria, the powerhouse of the cell, responsible for producing energy in the form of adenosine triphosphate (ATP). In the skeletal muscle, in addition to energy production, mitochondria are also involved in fatty acid oxidation and glycolysis. Moreover, mitochondria can adapt and proliferate in response to various stimuli. There is evidence suggesting that the impairment of mitochondria, induced by obesity, is a key factor contributing to the negative effects on skeletal muscle associated with obesity.

This review article will highlight the pivotal role that skeletal muscle mitochondria play in the context of obesity. We will discuss how obesity affects mitochondrial quality control systems in the skeletal muscle. We will then summarize current findings on caloric restriction and several dietary compounds and their implication as a therapeutic treatment against obesity-induced skeletal muscle mitochondrial dysfunction.

## Mitochondria in Skeletal Muscle

### Skeletal Muscle Fibers

Anatomically, skeletal muscle is structured as bundles of muscle fibers known as myofibers [3]. Each myofiber represents individual muscle cell, featuring the sarcomere as its fundamental functional unit. Bundles of myofibers form fascicles, which are covered by a layer of connective tissue known as perimysium. Bundles of fascicles together constitute muscle tissue, which is further wrapped around by a layer of endomysium [3].

Mammalian skeletal muscle fibers are heterogeneous in nature. They are categorized based on their contractile and metabolic characteristics, resulting in three major types of muscle fibers: slow-twitch oxidative fibers (Type I), fast-twitch oxidative fibers (Type IIA), and fast-twitch glycolytic fibers (Type IIB or IIX). Generally, each fiber type possesses a distinctive composition of contractile proteins influenced by several factors, including 1) myosin heavy chain (MHC) isoform, 2) contractile characteristics, and 3) calcium handling properties [4, 5]. On the other hand, the metabolic capacity of the muscle fiber depends on 1) capillary density, 2) mitochondrial content, and 3) insulin sensitivity [4].

Oxidative fibers have relatively low glycogen content but a high myoglobin content, imparting a distinctive red color to their appearance. These fibers feature the greatest number of mitochondria and capillaries, relying primarily on oxidative phosphorylation (OXPHOS) for ATP generation. This characteristic makes them exceptionally fatigue-resistant, ideally suited for endurance exercises (e.g., jogging, walking). In contrast, glycolytic fibers are characterized by a higher density of actin and myosin proteins, have less mitochondrial content, and appear white. They depend on anaerobic glycolysis for ATP generation, making them easily fatigable, and are best suited for short-duration, high-intensity exercises (e.g., jumping, weightlifting).

Despite the traditional classification of muscle fibers, it is important to recognize their adaptability to functional demands and substrate availability [6]. These adaptations are expected to result in a close connection between the contractile machinery and energy metabolism. For example, short-term high-fat diet feeding is attributed to an early adaptation toward slow-twitch, oxidative fiber types with increased OXPHOS complexes, likely in response to elevated fatty acids in circulation [7]. This adaptation, however, gradually decreases with prolonged high-fat diet exposure, ultimately resulting in fiber type shift towards glycolytic fibers and the eventual development of insulin resistance [8, 9]. It is noteworthy that mitochondria, as the primary players in OXPHOS, play a crucial role. Damage to mitochondrial DNA, for instance, results in changes in contractile properties [10], highlighting the regulatory role of mitochondria in determining skeletal muscle fiber type.

### Mitochondria in Skeletal Muscle

Mitochondria, often referred to as the powerhouse of the cell, are organelles enclosed by a double membrane with their own genetic material. Mitochondria exhibits a complex architecture consisting of four regions: the outer mitochondrial membrane (OMM), the intermembrane space, the inner mitochondrial membrane (IMM), and the matrix. The OMM contains porin channels that allow for the passage of ions, metabolites, and small molecules including its genetic materials, from the cytoplasm. Beneath the OMM lies the IMM, which forms cristae. Embedded within the IMM are various protein complexes involved in the electron transport chain (ETC) and ATP synthase, which collectively work to produce ATP through the transfer of electrons and the establishment of an electrochemical gradient. The mitochondrial matrix, enclosed by the IMM, houses enzymes and substrates essential for the tricarboxylic acid (TCA) cycle. The TCA cycle generates electron carriers, NADH and FADH_2_, that feed electrons into the ETC, facilitating ATP production.

Although mitochondria are commonly depicted as singular oblong structures, their shape varies significantly in different tissues. In skeletal muscle, for example, mitochondria form an interconnected network known as a reticulum, based on their location within the muscle fiber [11]. This unique feature of mitochondria in skeletal muscle is a result of the specific topology of energy utilization within elongated muscle cells, accounting for a dispersion of ATPases within myofibrils that span the entire length of the cell [12].

Mitochondria in skeletal muscle can generally be categorized into two subgroups based on their localization and morphology, with their shapes regulated by the metabolic demands of the muscle cell. Subsarcolemmal (SS) mitochondria are located underneath the sarcolemma membrane, close to the capillaries and nuclei. They have a globular shape [13] and play a crucial role in generating ATP for active membrane transport and gene transcription. Intermyofibrillar (IMF) mitochondria are localized in pairs between myofibrils near the Z-line of the sarcomere. They are elongated with a tubular shape extending between myofibrils [11, 13]. These IMF mitochondria primarily provide ATP to the contractile filaments in muscle, facilitating muscle contraction.

In the context of muscle fiber type, mitochondria can be vastly different in their quantitative and qualitative properties [14]. The quantity of muscle mitochondria reflects the relative importance of mitochondria to the energy requirement of each muscle fiber type. The qualitative properties of mitochondria include reactive oxygen species (ROS) production, structural relationship, and membrane properties, enzymatic stoichiometries, respiratory capacity, fuel selection, proton conductance [14]. Morphologically, mitochondria in oxidative fiber exhibit a grid-like mitochondrial network conformation, characterized by parallel and perpendicularly oriented elongated mitochondria. In contrast, mitochondria in glycolytic fiber appear more fragmented and are oriented perpendicular to the muscle contraction axis and the I band [15]. Furthermore, the mitochondrial connectivity within the network through IMFs is higher in oxidative fibers than in glycolytic fibers.

As mentioned previously, oxidative muscle fibers rely on mitochondria to provide most of the energy, whereas glycolytic fibers rely upon mitochondria to support basal and recovery metabolism. Thus, it is not surprising that obesity and insulin resistance contribute to muscle fiber type switch from oxidative to glycolytic type [9]. Indeed, impaired mitochondrial function directs fatty acids toward storage, as opposed to oxidation, which contributes considerably to intramyocellular lipid accumulation and insulin resistance [16]. Readers interested in deeper understanding of the mitochondrial specialization in different skeletal muscle fibers and their physiologic relation to skeletal muscle health can refer to an outstanding review by Dong and Tsai [17].

## Mitochondrial Quality Control

Mitochondria face constant challenges from the production of ROS, rendering them especially susceptible to DNA mutations and protein misfolding. Notably, skeletal muscle experiences an elevated ROS production due to cellular responses to elevated levels of free fatty acids and hyperglycemia in obesity [18, 19]. Ultimately, this dysfunction leads to decreased mitochondrial content, decreased genetic material, and decreased rate of OXPHOS [19, 20]. Thus, mitochondrial dysfunction in the liver, adipose tissue, and skeletal muscle has been the subject of extensive research. In this context, an intricate network of quality control machinery plays a vital role in safeguarding mitochondrial integrity, a process critically linked to numerous human diseases, such as obesity [19], diabetes [21], and age-related conditions [22, 23], all of which can lead to skeletal muscle atrophy [24]. While a comprehensive investigation on the role of mitochondrial dysfunction in skeletal muscle atrophy is beyond the scope of this review, it has been extensively discussed by Chen et al. [24].

Mitochondria are highly dynamic organelles that are constantly undergoing synthesis and recycling processes essential for sustaining optimally functioning pool of mitochondria [11]. The morphology of mitochondria is complex in various cell types, giving rise to a network of IMM and OMM that extend over long distances within the cytoplasm of the cell. This extended mitochondrial reticulum, with any signs of dysfunction, can undergo fragmentation into smaller organelles as a preparation step for autophagic removal and recycling. This autophagic elimination of mitochondria is achieved either non-selectively through general autophagy, or selectively, through specific, mitochondria-targeting autophagy, known as mitophagy [25]. This elimination process is part of mitochondrial quality control mechanisms, which plays an important role in preserving mitochondrial integrity and function.

### Mitophagy

**Fig. 1:**
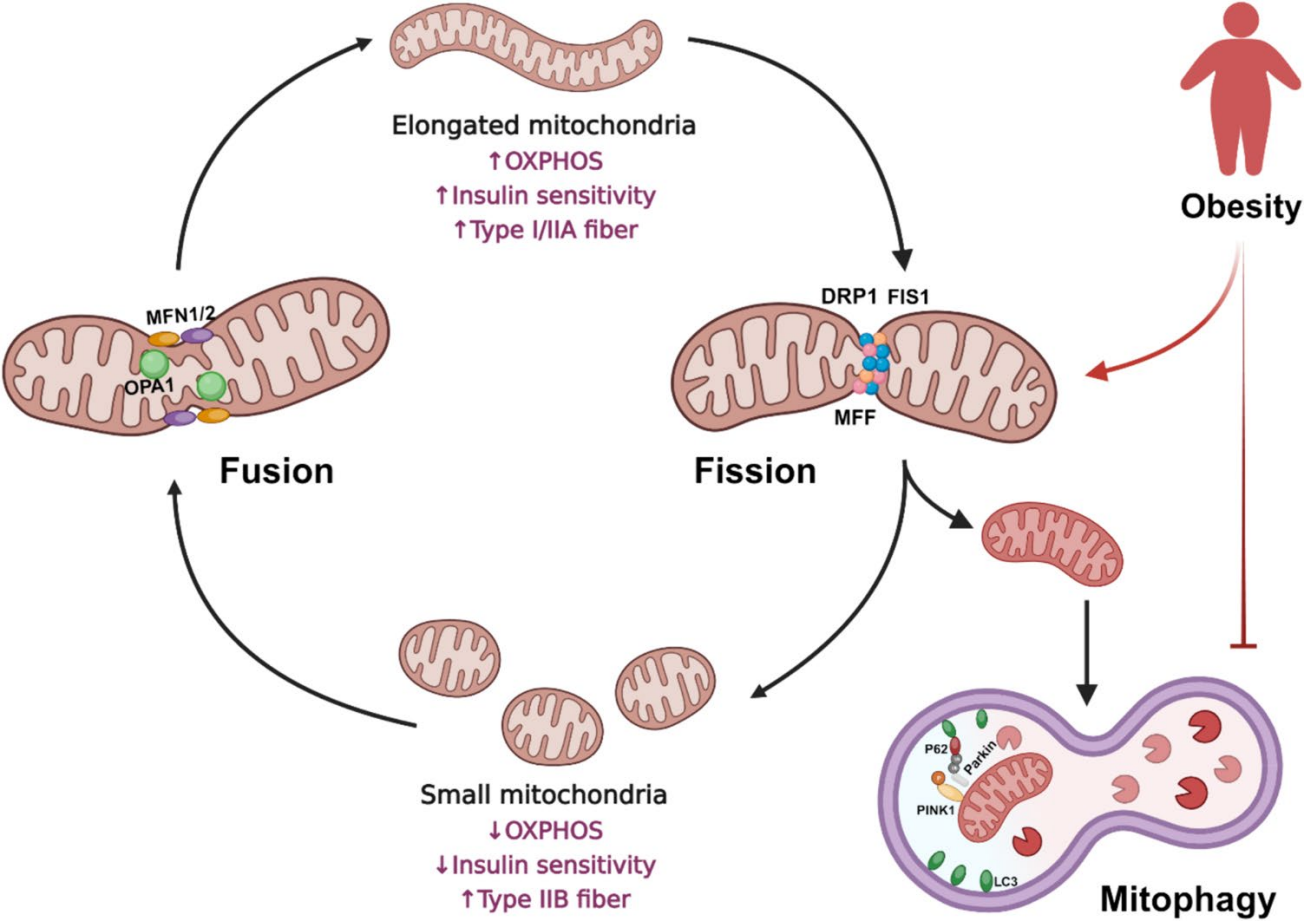
Schematics of mitochondrial dynamics and mitophagy. The fusion of multiple mitochondria involves MFN1/2 and OPA1 proteins. Elongated mitochondria are predominant in type I and IIA muscle fibers, contributing to increased insulin sensitivity and OXPHOS. Obesity induces the fission of mitochondria into smaller fragmented organelles, resulting in diminished insulin sensitivity and OXPHOS, ultimately leading to muscle fiber type switch towards glycolytic fibers. This fission process involves DRP1, FIS1, and MFF proteins. PINK1-Parkin mediated mitophagy involves accumulation of PINK1 on the OMM, followed by recruitment of Parkin and ubiquitination of various OMM proteins. The ubiquitinated mitochondria is then engulfed by an autophagosome via p62, connecting ubiquitin to LC3B for autophagy destruction. Obesity inhibits mitophagy, leading to accumulation of dysfunctional mitochondria. Created with BioRender.com

The most studied pathway for mitophagy in skeletal muscle involves PTEN-induced putative kinase 1 (PINK1) and Parkin (Fig. 1). Various stressors, including the loss of mitochondrial membrane potential, excessive ROS production, and mutations in mitochondrial DNA (mtDNA), can activate PINK1 activity [12]. Briefly, as the mitochondrial organelle loses its membrane potential, PINK1 accumulates on the OMM. The autophosphorylation of PINK1, followed by subsequent phosphorylation of ubiquitin, recruits the E3 ligase Parkin, which, in turn, polyubiquitinates various OMM proteins. These ubiquitin chains serve to tether the targeted mitochondria to the autophagosome via adaptor proteins, such as sequestosome-1 (SQSTM1/p62), BRCA1 gene 1 protein (NBR1), nuclear dot protein 52 (NDP52), and optineurin (OPTN), connecting ubiquitin to microtubule-associated protein 1A/B-light chain 3 (LC3)-II present in the autophagosome [12]. This engulfment of mitochondria by autophagosome ultimately leads to their destruction.

This degradation system serves to prevent the vicious cycle of oxidative stress and mitochondrial damage. Appropriate mitophagy, under basal condition, prevents the accumulation of damaged and dysfunctional mitochondria and allows maintenance of healthy mitochondria pool. Dysregulated mitophagy, however, can impact the mitochondrial quantity and function [19]. While previous reports remain conflicting, there are evidence suggesting a disruption in mitophagy in the skeletal muscle in obesity [26], long-term high-fat diet consumption [27], and aged skeletal muscle [22, 23]. Regarding obesity, for instance, Greene et al. reported decreased mitophagy in obese animals with accumulation of dysfunctional organelles [26]. Conversely, earlier studies reported accumulated markers of mitophagy in the muscles of high-fat diet-fed mice [27]. However, this observation remains debated as many of these early studies did not accurately measure the rate of autophagic flux. Indeed, later studies showed that lipotoxicity associated with obesity inhibits the autophagic flux and defective degradation is the main cause of autophagosome accumulation in the tissues of obese model [28]. Nevertheless, future studies are needed to elucidate the complex nature of mitochondrial reticulum remodeling and the precise regulation of mitophagy in skeletal muscle of obese phenotype in response to diverse metabolic factors.

### Mitochondrial Biogenesis

Mitochondrial biogenesis can be defined as the growth and division of pre-existing mitochondria. This fundamental process maintains the mitochondrial content within organs as these organelles cannot be generated de novo. With their own genome capable of auto-replication, mitochondria rely on the coordinated synthesis and import of approximately 1,000 to 1,500 proteins encoded by both nuclear and mitochondrial genomes. mtDNA is a double-stranded circular molecule of about 16.5 kb, consisting of 37 genes encoding 13 subunits of the ETC complexes I, III, IV, and V. Various environmental stressors such as exercise, caloric restriction, low temperature, oxidative stress, cell division, renewal, and differentiation impact the dynamics of mitochondrial biogenesis. A more comprehensive discussion of these regulatory factors is available in a review by Jornayvaz and Shulman [29].

### Regulation of Mitochondrial Biogenesis

Several proteins intricately regulate mitochondrial biogenesis, responding to various physiological stimuli such as physical activity, diet, temperature, and muscle myogenesis [30]. Peroxisome proliferator-activated receptor (PPAR) γ coactivator 1α (PGC1α) lies at the center of mitochondrial biogenesis, interacting with diverse transcription factors and proteins crucial for mitochondrial oxidation capacity and internal mitochondrial function [31]. PGC1α is widely distributed in various organs and tissues, particularly in skeletal muscle and brown adipose tissue which are rich in mitochondria [32]. Activation of PGC1α in skeletal muscle has been shown to regulate carbohydrate and lipid metabolism and to improve oxidative capacity of the muscle fiber by inducing mitochondrial biogenesis [33].

**Fig. 2:**
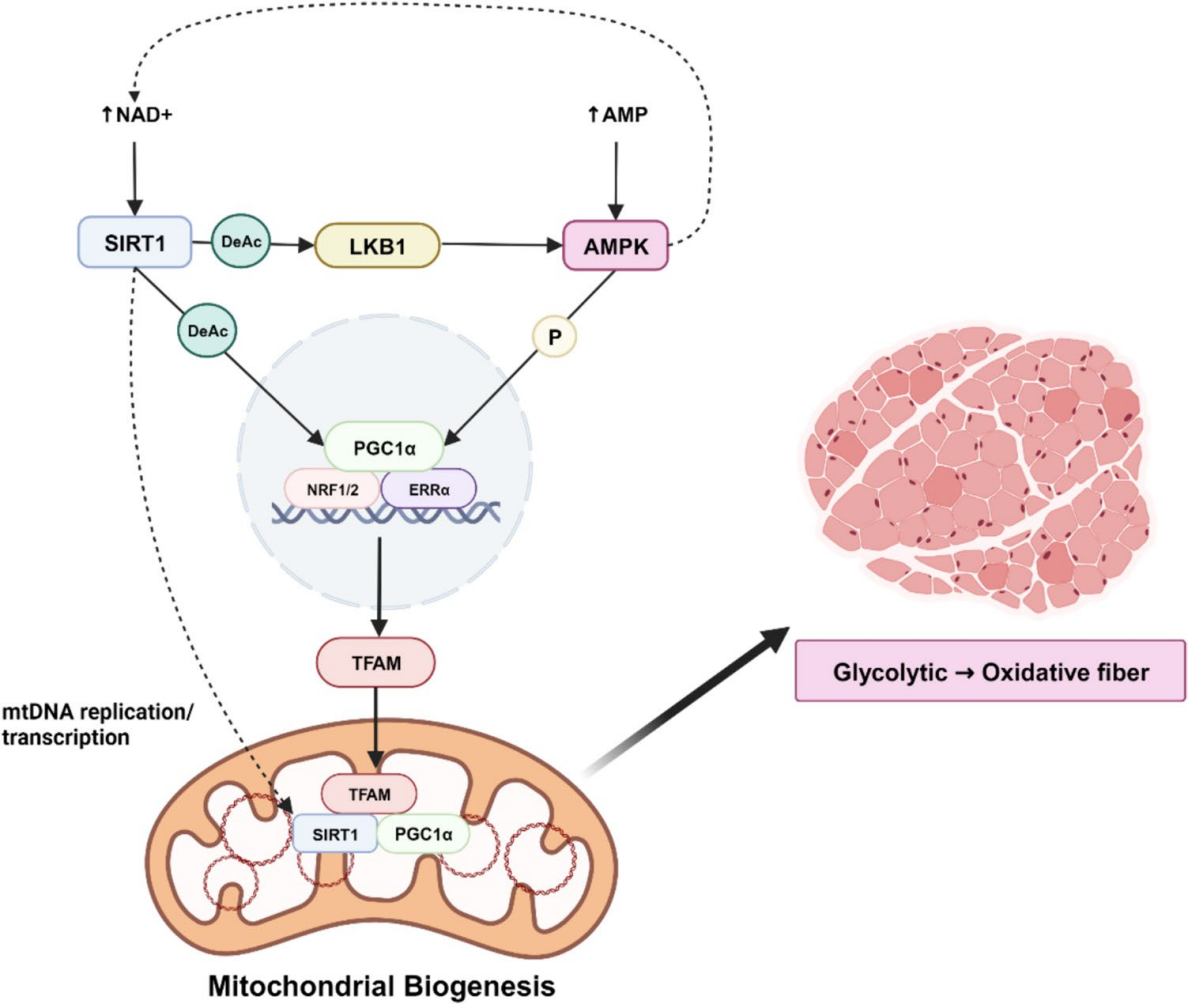
Diagram of mitochondrial biogenesis pathways. Elevated cellular levels of NAD^+^ and AMP activate SIRT1 and AMPK, respectively. SIRT1 activates both AMPK and PGC1α by deacetylation, while AMPK activates PGC1α by phosphorylation and SIRT1 by increasing NAD^+^ levels. In the nucleus, PGC1α activates NRF1/2 and ERRα, subsequently leading to TFAM activation. TFAM then translocate to the mitochondria, influencing mtDNA replication and transcription, resulting in mitochondrial biogenesis. Increased mitochondrial biogenesis has been associated with muscle fiber type shift towards oxidative fibers. Created with BioRender.com

Various stimuli influence the translocation of PGC1α into the nucleus, where it exerts its transcriptional activity. Once inside the nucleus, PGC1α coactivates numerous transcription factors, including estrogen-related receptor α (ERRα), nuclear respiratory factor 1 and 2 (NRF1 and NRF2), and PPARγ [32] These transcription factors regulate the expression of many nuclear-encoded mitochondrial genes including mitochondrial transcription factor A (TFAM) and components of the respiratory chain (COX subunits) [25]. Subsequently, TFAM mediates mtDNA replication and transcription, thereby enhancing mitochondrial biogenesis [32]. While there are several upstream regulatory factors influencing PGC1α activities through transcription and posttranslational modification, including AMPK, SIRT1, CREB, and p38 mitogen-activated protein kinase (MAPK) [32, 34] (Fig. 2), this review discusses the two gatekeepers of PGC1α in skeletal muscle, AMPK and SIRT1.

5’-Adenosine monophosphate-activated protein kinase (AMPK) is a Ser/Thr kinase, existing as a heterotrimeric complex consisting of catalytic α (α1 and α2) and regulatory β (β1 and β2) and γ (γ1, γ2, and γ3) subunits. In skeletal muscle, AMPK acts as an important regulator of energy metabolism, impacting glucose uptake, lipid oxidation, and mitochondrial biogenesis [35]. Under conditions of increased intracellular AMP/ATP ratio, AMP or ADP binds to the γ subunit of AMPK, inducing conformational changes and enhancing phosphorylation. This activation restores intracellular energy levels through a cascade of events, including the phosphorylation of its downstream targets, such as PGC1α. AMPK can also regulate PGC1α indirectly through AMPK-dependent p38 MAPK [36].

While traditionally regarded as a catabolic agent in skeletal muscle, recent studies revealed the dual nature of AMPK in both muscle development and growth regulation. Specifically, phosphorylation of AMPKα1 subunit at Thr183 plays a prominent role in stimulating anabolism during regeneration process, while phosphorylation of AMPKα2 subunit at Thr172 plays a role in regulating muscle degradation during atrophy [37]. Notably, skeletal muscle-specific AMPKα1α2 double-knockout (mdKO) mice manifest impaired endurance exercise capacity [35]. Intriguingly, these mice show diminished maximal ADP-stimulated mitochondrial respiration without alterations in mitochondrial numbers, highlighting AMPK’s role in regulating mitochondrial oxidative capacity and substrate utilization without influencing the baseline mitochondrial content. Although the specific isoform of AMPKα subunit was not investigated, this regulation contributes significantly to the maintenance of skeletal muscle integrity [35].

A metabolic sensor and protein deacetylase, silent information regulator 2 (Sir2) homologue Sirtuin, stands as a gatekeeper in the regulatory network of metabolic homeostasis. The family of Sirtuin has been highly recognized for its role in anti-aging, prolonging life span, and regulating metabolism [38]. Sirtuins need to interact with nicotinamide adenine dinucleotide (NAD^+^) to induce physiological function and mediate the deacetylation of target substrate [32]. From the several isotypes (SIRT1-7), SIRT1 engages in diverse enzymatic activities in the nucleus, cytoplasm, and mitochondria [32, 39]. In skeletal muscle, SIRT1 promotes deacetylation of liver kinase B1 (LKB1), which triggers AMPK activation. AMPK and SIRT1 are known to reciprocally regulate each other as they share many common target molecules. For instance, activation of AMPK by SIRT1 increases cellular NAD^+^ levels and further induces SIRT1 activation [32, 40]. SIRT1 can also directly activate TFAM in coactivation with activated PGC1α. TFAM then transports SIRT1 and PGC1α into mitochondria, forming a complex with the D-loop region of mtDNA, thereby influencing mitochondria biogenesis [32, 39]. Notably, SIRT1 transgenic muscle exhibits a shift from fast-to-slow twitch fiber types, increased PGC1α levels, and decreased expression of muscle atrophy markers [41].

### Mitochondrial Fusion

Mitochondria are highly dynamic organelles, actively engaging in a coordinated cycle of fusion and fission processes, collectively known as “mitochondrial dynamics”. These processes are widely recognized as key factors contributing to both mitochondrial and cellular health [42]. The mitochondrial fusion process merges smaller mitochondria in a cell, creating an elongated and more interconnected mitochondria. During this process, the OMM and the IMM merge, facilitating the exchange and redistribution of mitochondrial contents, including mtDNA, proteins, lipids, and metabolites between the merging organelles. Notably, fusion events act as a quality control mechanism, allowing damaged or dysfunctional mitochondria to mix with healthy counterparts [43]. In the context of skeletal muscle, several key players involved in this process include the GTPase transmembrane proteins mitofusins (MFN1 and MFN2) anchoring adjacent OMM and the optic protein atrophy 1 (OPA1) responsible for fusing the IMM [44].

Emerging studies underscore the importance of mitochondrial dynamics in skeletal muscle physiology. Fusion events optimize maximal oxidative capacity, mitigating cellular stress by intermixing contents of partially damaged mitochondria. Moreover, fusion between mitochondria is proposed as a mechanism to rescue mutated mitochondria through cross-complementation, lessening the impact of environmental damage [45]. Notably, cells engineered to contain a mix of wild-type and pathogenic mitochondria remain functional until the proportion of pathogenic mitochondria surpasses a critical threshold [45]. This protective mechanism of mitochondrial fusion, prior to reaching the critical threshold, primarily operates through stabilizing mtDNA copy number and preventing the accumulation of mtDNA mutations [45]. Indeed, conditional deletion of *Mfn1* and *Mfn2* results in severe mtDNA depletion in muscle and causes severe mitochondrial dysfunction, compensatory mitochondrial proliferation, and muscle atrophy [45]. As result of mtDNA depletion, studies with knockout models also show a progressive decline in exercise performance, accompanied by impaired electron transport chain, particularly for complex I and IV [43].

Notably, mitochondrial fusion responds to the metabolic state of the muscle fiber, and thus, distinct muscle fiber types exhibit characteristic mitochondrial fusion patterns. Oxidative muscle fibers, such as type I and IIA, contain elongated mitochondrial networks with heightened fusion rates that are dependent on MFN1 and MFN2 [43]. Furthermore, switching of a glycolytic fiber to an oxidative IIA type correlate with elongation of mitochondria, suggesting a link between mitochondrial fusion and metabolic states [43]. In skeletal muscle, MFN2 appears to play a particularly important role. Skeletal muscle exhibits a high abundance of MFN2, and disruption of this protein have been related to disruption in mitochondrial membrane potential and cellular oxygen consumption [46]. Mice with skeletal muscle-specific deletion of *Mfn2* exhibit impaired mitochondrial morphology, localization, and calcium uptake, ultimately resulting in decreased muscle cross-sectional area and strength. Nie et al. reported that *Mfn2*-deficient mice display impaired insulin sensitivity attributed to elevated oxidative stress [47]. In L6 cells treated with palmitate and small interfering RNA (siMfn2), repression of *Mfn2* mRNA expression coincides with reduced mitochondrial membrane potential and antioxidant enzyme activities, resulting in elevated ROS production and the phosphorylation of inflammatory cytokines. Upon reintroduction of MFN2, the oxidative stress and insulin resistance are alleviated [47].

### Mitochondrial Fission

Mitochondrial fission is a complex, multi-step process in which a single mitochondrion divides into two or more smaller organelles, each containing its own membranes and contents. The critical regulator of this intricate process is the cytosolic GTPase dynamin-related protein 1 (DRP1). The fission initiates with mitochondria establishing contact with the endoplasmic reticulum (ER), a crucial player in identifying the scission site within the mitochondrial network [48]. During the initial phase, fission takes place at mitochondria-ER contact sites, marked by mtDNA. At these sites, ER tubules enwrap the mitochondria, initiating a reduction of mitochondrial diameter—a process termed ER-associated mitochondrial division (ERMD). Subsequently, DRP1 is recruited to these marked division sites, binding to its OMM receptors and adaptors, namely mitochondrial fission factor (Mff) and fission protein 1 (Fis1). This binding event facilitates the oligomerization of DRP1, leading to the formation of a ring-like structure that promotes complete division of the mitochondria [15].

The division of mitochondria results in the segregation of mitochondrial contents, including mtDNA, proteins, lipids, and metabolites. Consequently, this fission process enhances mitochondrial diversity within a cell, facilitating adaptation to varying cellular energy demands and responses to stress or damage. Furthermore, mitochondrial fission is another important quality control mechanism, isolating damaged or dysfunctional mitochondrial segments for removal by mitophagy [45]. This process, in conjunction with mitochondrial fusion, ensures the dynamic and responsive nature of the mitochondrial network in cells, optimizing energy production and adapting to ever-changing physiological conditions.

In the context of skeletal muscle, atrophic or catabolic conditions prompt a remodeling of the mitochondrial network. Of particular importance, even a mild imbalance between fusion and fission has been demonstrated to impair skeletal muscle mass [49]. For instance, overexpression of the fission proteins, or certain metabolic conditions inducing mitochondrial fission, adversely impacts skeletal muscle development and repair [49], leading to muscle atrophy [50]. Romanello et al. reported that co-transfecting mitochondria with DRP1 and FIS1 induces alterations in mitochondrial morphology and activates autophagy [50]. Over time, the sustained level of DRP1/FIS1 contributes to a reduction in muscle fiber size and muscle atrophy [50].

## Impact of Obesity on Skeletal Muscle Mitochondrial Systems

Skeletal muscle from obese individuals demonstrates muscle fiber type shift from oxidative- (Type I/IIA) to glycolytic-type (Type IIB) [9]. This shift, particularly the growth of type II fiber, is a protective mechanism to sustain metabolic homeostasis, which is mediated by Brg1/Brm-associated factor (Baf60c) [51]. The Bag60c pathway promotes Akt activation, improving glucose tolerance and insulin sensitivity. The activation of Akt also leads to hypertrophy of type II muscle fibers, further impacting the fiber type proportion. Additionally, fiber type transition can also largely depend on the skeletal muscle mitochondrial oxidative capacity. As such, it has been suggested that the increased accumulation of intramuscular lipids in obese individuals results from reduced fatty acid oxidation, which is caused by metabolic inflexibility due to a diminished type I muscle fibers [52]. This metabolic inflexibility limits the utilization of lipids for energy in skeletal muscle. Consequently, reduced mitochondrial oxidative capacity further impairs skeletal muscle oxidation, contributing to the development of insulin resistance commonly associated with obese individuals [53]. Therefore, the alteration in fiber type proportion impacts whole body metabolism and accelerates the development of insulin resistance. This review will explore how obesity affects metabolism in skeletal muscle by disrupting mitochondrial biogenesis and dynamics systems, which are critical for maintaining metabolic health.

### Mitochondrial Biogenesis in Obesity

In the context of mitochondrial biogenesis regulators, lower levels of mitochondrial biogenesis markers, AMPK, SIRT1, and PGC1α, are detected in the muscles of obese animals [54, 55]. These levels are accompanied by significant decreases in the expression of genes related to mitochondrial biogenesis (*Nrf*, *Tfam*, *Pgc1α*, and *Sirt1*), fatty acid oxidation, and ETC complex subunits, indicating the effect of obesity on mitochondrial function. Notably, obesity-resistant lean phenotype tends to have a higher expression of mitochondrial biogenesis markers, indicating potential relationship between mitochondrial biogenesis in skeletal muscle and the onset of obesity and obesity-resistance development in the course of high-fat diet feeding [56].

Obesity inhibits AMPK activity, which negatively impacts PGC1α transcription and its co-activator functions, [57] thereby impairing mitochondrial biogenesis in skeletal muscle. Impact of obesity on skeletal muscle mitochondrial biogenesis signaling pathway can hinder muscle fiber type proportions. PGC1α is highly expressed in skeletal muscle, particularly in oxidative slow muscle fibers, which explains why obese individuals exhibit lower oxidative fiber proportion. In this regard, overexpression of PGC1α promotes glycolytic-to-oxidative fiber-type shift and mitochondrial biogenesis in skeletal muscle [58]. In agreement, skeletal muscle-specific knockout of PGC1α promotes muscle fiber type shift from oxidative to glycolytic [33]. Thus, AMPK/PGC1α may be an important therapeutic target to preserve oxidative fibers and insulin sensitivity in skeletal muscles of obese individuals. Notably, resveratrol, one of the well-known bioactive polyphenols and popular exercise mimetic, protects against obesity-induced impairments in mitochondrial biogenesis by stimulating SIRT1/AMPK/PGC1α pathway [57].

Mitochondrial biogenesis is critical for myogenic differentiation and muscle regeneration [57]. In this regard, recent studies have shed light on the impact of obesity during myogenic differentiation and muscle regeneration. High-fat diet-induced obese mice exhibited decreased mtDNA content, expression of PGC1α, and several mitochondrial markers. Moreover, AMPK activity was downregulated in the muscles of obese animals, leading to attenuated muscle regeneration. This was confirmed when activation of AMPK increased the density of quiescent satellite cells, enhanced satellite cell proliferation, and promoted satellite cell myogenic differentiation in regenerating muscle [57]. This AMPK-mediated muscle cell regeneration is supported by the fact that AMPKα1 catalytic subunit potentiates myogenic expression, myogenesis, and muscle regeneration [59].

In summary, obesity impacts mitochondrial metabolism, primarily through the adjustment of mitochondrial biogenesis markers induced by intramuscular lipid accumulation. This impact manifests in a preference for glycolytic fiber types. Targeting the key mitochondrial biogenesis molecules, such as PGC1α, SIRT1, and AMPK, holds promise for understanding their role in preserving mitochondrial function and potentially mitigating the undesired shift in muscle fiber types in obese individuals. Further investigation into these molecular pathways may offer valuable insights into potential interventions to counteract obesity-induced muscle fiber type switch.

### Mitochondrial Dynamics and Obesity

Regulation of mitochondrial fusion and fission play a crucial role in maintaining a healthy pool of mitochondria within skeletal muscle. Disruptions in mitochondrial dynamics can lead to aberrant mitochondrial morphology, compromised respiratory function, and decreased ATP content in skeletal muscle [60]. Notably, previous research highlights the significance of dysfunctional mitochondrial dynamics in both obese animal [26, 61] and human [62] models.

In the context of morbid obesity, elevated fasting plasma free fatty acids correlate with mitochondrial fragmentation [62]. This is attributed to the downregulation of fusion proteins (both MFN1 and MFN2) and upregulation of fission protein (DRP1) [62]. Similar abnormalities in mitochondrial fusion and fission proteins were also demonstrated in the muscles of obese zebrafish and high-fat diet-fed obese rats. These obese animals exhibit higher expression levels of DRP1 and lower levels of OPA1 and MFN2 [55, 61]. Moreover, the disrupted mitochondrial dynamics results in morphological changes such as degeneration, enlargement, and swelling of mitochondria with disrupted cristae [55].

Previous studies showed that insulin-resistant models typically exhibit a reduced fusion-to-fission ratio, generally accompanied by more fragmented, less efficient, and ROS-producing mitochondria. The connection between skeletal muscle mitochondrial dysfunction and insulin resistance is complex [63, 64], but it is generally accepted that alterations in mitochondrial structure and function modulate intraorganellar communication [64]. This, in turn, leads to novel endocrine responses in muscle cells, influencing systemic metabolic homeostasis [64]. Notably, fragmented mitochondria in skeletal muscle (and liver) have been linked to the decreased aerobic capacity and development of insulin resistance [65], suggesting that protein involved in mitochondrial dynamics play a regulatory role in systemic metabolism. In this regard, previous study demonstrated that increasing fusion-to-fission ratio through genetic and pharmacologic DRP1 inhibition could modify fatty acid-induced mitochondrial fragmentation, depolarization, and insulin resistance in C2C12 cells [66]. Additional evidence supports the negative effect of decreased mitochondrial fusion on insulin sensitivity in skeletal muscle. For instance, MFN2-knockout mice exhibited enhanced hepatic glucose production and impaired response to insulin [67, 68]. The transcriptional activity of *Mfn2* gene was shown to be stimulated by PGC1α, as overexpression of PGC1α enhanced *Mfn2* mRNA and protein levels in cultured muscle cells and reduced PGC1α expression in obese and diabetic muscle repressed MFN2 level [46], emphasizing the role of PGC1α as regulators of mitochondrial morphology and insulin sensitivity.

Interestingly, discrepancies arise in different studies, with some reporting no alteration of MFN1/2 and OPA1 proteins in palmitic acid-treated C2C12 cells and in obese mice fed a high-fat diet for 10 weeks [66]. In contrast, another study reported lower levels of both fission and fusion proteins (FIS1 and OPA1, respectively) in obese individuals compared to endurance-trained athletes [65]. These varying results may be attributed to differences in diet duration and body weight changes [66], suggesting that fusion and fission processes occur at distinct time intervals. Moreover, it is essential to standardize the normalization of these proteins across studies to avoid discrepancies [65]. For instance, in a study by Kristensen et al., mitochondrial dynamics proteins were normalized to citrate synthase activity, a marker of mitochondrial content [62], while other studies quantified mRNA expression or normalized protein levels to GAPDH [60]. While direct assessments of mitochondrial dynamics pose challenges, necessitating advanced techniques such as time-lapse microscopy [26], future investigations should strive to define the balance of mitochondrial dynamics in obesity. This is crucial for illuminating the direct impact of obesity on mitochondrial fission and fusion dynamics, considering the significant role these dynamics play in the development of metabolic diseases, particularly insulin resistance.

## Diet as a Therapeutic Approach

**Fig. 3:**
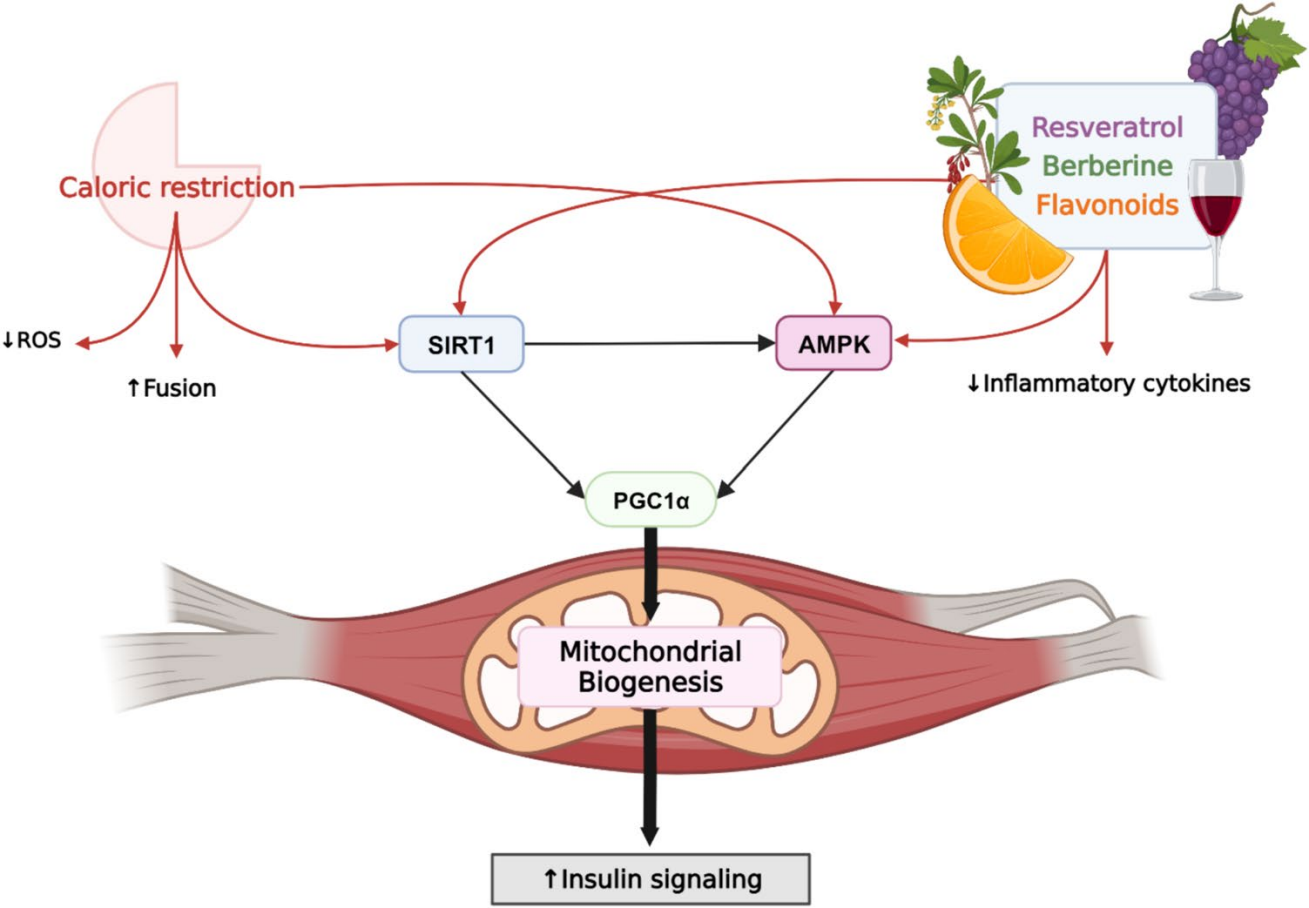
Schematics of the effects of caloric restriction and nutraceuticals on mitochondrial biogenesis. Caloric restriction activates SIRT1, which activates both AMPK and PGC1α, leading to mitochondrial biogenesis and increased insulin signaling. Caloric restriction enhances mitochondrial fusion in oxidative muscle fibers. Nutraceuticals such as resveratrol, berberine, and flavonoids enhance mitochondrial biogenesis through the activation of SIRT1 and AMPK. Both caloric restriction and these nutrients contribute to preservation of mitochondrial quality by reducing levels of ROS and inflammatory cytokines. Created with BioRender.com

Non-pharmacological interventions have gained significant interest in the prevention of various metabolic diseases, with caloric restriction and natural bioactive compounds found in foods taking limelight in research. Recently, caloric restriction and several nutraceuticals have been explored for their potential to modulate mitochondrial function and as a promising therapeutic approach. A common theme emerging from studies is that caloric restriction and these natural compounds elicit changes in cellular mitochondrial content and mitigates mitochondrial ROS production [69]. Notably, resveratrol is often referred to as a caloric restriction mimetic, as it has been shown to extend lifespan through mechanisms similar to those associated with caloric restriction. While this review briefly highlights caloric restriction, resveratrol, and a few other natural compounds (Fig. 3), more comprehensive reviews on dietary bioactive compounds that impacts mitochondrial health are available elsewhere for the readers interested in more in-depth information [69].

### Caloric Restriction

One of the most popular non-pharmacological interventions known to promote longevity and protect against metabolic diseases is caloric restriction. Moderate reduction of calorie intake to 30–50% less than ad libitum without malnutrition can delay the onset of age-related diseases, improve stress resistance, and slow functional decline [70]. While the precise mechanisms remain to be elucidated, these effects are suggested to occur through delaying mitochondrial aging, and by increasing mitochondrial biogenesis and remodeling. This remodeling makes mitochondria more efficient – increased proportion of respiration coupled to ATP production and reduced overall oxygen consumption and producing less ROS [71], among the various mechanisms by which caloric restriction might act, SIRT1 has been the focus of much attention because of its ability to integrate sensing of the metabolic status with adaptive transcriptional outputs. Notably, deacetylase activity of SIRT1 in skeletal muscle is increased in animals on a 20-day caloric restriction diet (60% of ad libitum intake), concomitant with enhanced insulin signaling and glucose uptake [72]. In another study, middle-aged rats on 14 weeks of 40% caloric restriction displayed lower glucose metabolism enzymes than the ad libitum group [73], implicating metabolic pathway reprogramming from glycolysis to OXPHOS. In addition, markers of mitochondrial biogenesis, including AMPK and SIRT1, and COXIV content, were increased in the caloric restriction group [73]. This finding also explains the association between increased AMPK activation, decreased lactate levels, increased NAD^+^ abundance, and an enhanced NAD^+^/NADH ratio, ultimately leading to upregulation of SIRT1 [73].

According to a study by Civitarese et al., 6 months of 25% caloric reduction improves skeletal muscle mitochondrial biogenesis markers [74]. However, mitochondrial content and enzyme activities remained unchanged, with the authors proposing that caloric restriction induces the proliferation of mitochondria with “efficient” ETC systems coupled with lower oxygen consumption [74]. Similarly, a separate study reports that a 36 weeks of 60% caloric restriction enhances the transcription levels of genes involved in ROS scavenger function and energy metabolism without altering mitochondrial function and enzyme activities [75]. Hempenstall et al. corroborated these findings, observing increased levels of PGC1α, several mitochondrial associated proteins, and cytochrome *c* oxidase content in skeletal muscle of 30% caloric restriction mice [76]. Nevertheless, caloric restriction does not alter mitochondrial content in these mice, determined by citrate synthase activity [76].

Conversely, a study by Lanza et al. showed that life-long 40% caloric restriction preserves mitochondrial capacity and efficiency in aged mice without substantially affecting mitochondrial biogenesis [70]. This study, however, highlighted the effect of chronic caloric restriction in preserving mitochondrial function by maintaining the integrity of protein and DNA, reducing mitochondrial oxidant emission, and promoting endogenous antioxidant activity [70]. The discrepancies among these studies may stem from differences in the interpretations of transcriptional regulation of mitochondrial biogenesis markers, as mRNA abundance and protein expression may not always align [70]. Moreover, subcellular localization of PGC1α should be kept in mind, as its location determines its function. For example, PGC1α is found in both nucleus and cytoplasm under basal conditions but translocate into the nucleus upon stress stimuli and initiates transcriptional activities [73].

Regarding energy status, caloric restriction can have a significant impact on weight loss and reduction of intramuscular fat accumulation through whole-body lipid oxidation [77]. This, in turn, can improve mitochondrial health by mitigating lipid-induced oxidative stress. Whether the effects of caloric restriction on mitochondria is simply through weight loss is unclear as weight loss itself can enhance OXPHOS efficiency in muscle of obese animals concomitant to a decrease in whole-body energy expenditure [78]. Notably, weight loss in obese mice did not robustly alter skeletal muscle mitochondrial proteome [78]. The effects of caloric restriction may be due to several factors, including its ability to increase uncoupling proteins which may ameliorate oxidative damage by reducing reactive oxygen species [79]. Conversely, the effects of caloric restriction on muscle mitochondrial function in non-obese individuals remain inconclusive [77]. In a study by Sparks et al., only those subjects with a baseline of “more coupled” mitochondria benefited from caloric restriction, with improvements in muscle mitochondrial ATP synthesis rates and coupling [77]. Nonetheless, caloric restriction is effective in ameliorating obesity-related metabolic impairments and increasing mitochondrial biogenesis markers, primarily through metabolic pathways involving AMPK and SIRT1 [80].

The mechanisms of caloric restriction on skeletal muscle mitochondria are complex. While previous findings indicate that caloric restriction impacts mitochondrial biogenesis markers, whether it enhances mitochondrial content and function remains controversial. It seems that caloric restriction induces an adaptive response in PGC1α, increasing mitochondrial respiration and reducing oxidative stress, thereby maintaining healthy mitochondrial population. Caloric restriction research should start with careful planning, considering various factors such as age [76], subcellular fractionization of samples for investigating PGC1α activation, and variations in the length of the study period, all of which can significantly impact study results.

### Resveratrol

Resveratrol is one of the most studied polyphenolic compounds mainly found in the skin and seed of grapes, peanuts, and other plants. Widely recognized for its health benefits, as often implicated in the “French paradox”, resveratrol triggers a plethora of biological responses in cells and organisms, encompassing various adaptive mechanisms. A key property of resveratrol is the activation of SIRT1, enhancing SIRT1-dependent cellular processes such as axonal protection, fat mobilization, and inhibition of NFκB-dependent transcription [81]. In yeast, resveratrol has demonstrated its capacity to extend lifespan, implicating the potential of resveratrol as an anti-aging agent [81] with potential clinical implications. In skeletal muscle, resveratrol can prevent muscle atrophy in several catabolic conditions, such as cancer, diabetes, chronic kidney disease, and disuse [61].

Recent studies have also revealed anti-obesity effects of dietary resveratrol supplementation [82, 83], potentially by mimicking the metabolic effects of long-term caloric restriction [84] and improving adipose tissue function and insulin sensitivity in obese model [85]. In animal studies, resveratrol treatment increases mitochondrial content and prevents alterations in mitochondrial morphology in the muscles caused by high-fat diet (in 0.4% concentration for ten weeks) [61] and diabetic condition (in 0.04% concentration for eight weeks) [86]. The preservation of mitochondrial morphology by resveratrol treatment was associated with improved mitochondrial bioenergetics, evidenced by enhanced mitochondrial membrane potential, activities of mitochondrial respiratory chain complex, and ATP content in skeletal muscle [61]. Ultimately, the effect of resveratrol in preserving mitochondrial morphology and bioenergetics led to hypertrophy of mitochondria-rich type I and IIA muscle fibers [86].

In a placebo-controlled, double-blind cross-over study, 150 mg/day of resveratrol treatment for 30 days significantly reduced blood glucose and insulin concentrations in otherwise healthy obese men [84]. In an animal study, 100 mg/kg of daily resveratrol treatment for eight weeks protected against high-fat diet-induced intramuscular lipid accumulation and insulin resistance and reverts the decline in SS mitochondrial β-oxidation [87]. In this study, however, high-fat diet and resveratrol treatment did not have any effect on IMF mitochondria. This is not surprising because SS mitochondria are the main energy provider related to substrate oxidation and insulin action, while IMF mitochondria play a more direct role in supporting muscle contractions [88]. Moreover, the close proximity of SS mitochondria to the sarcolemma membrane facilitates their interaction with key proteins involved in the insulin signaling cascade more effectively than IMF [88].

Studies have demonstrated the characterization of molecular targets of resveratrol, including PGC1α, SIRT1, NFκB, AMPK, FoxO3, and PPARγ [16, 61, 85, 89], that are responsible for modulating mitochondrial activity. In the muscle biopsy of healthy obese males supplemented with resveratrol, increased AMPK phosphorylation, SIRT1 and PGC1α expression, and citrate synthase activity were reported [84]. These results indicate that resveratrol influences mitochondrial biogenesis directly via SIRT1/AMPK/PGC1α signaling pathways. Additionally, resveratrol up-regulates the expression of genes associated with mitochondrial oxidative phosphorylation, while down-regulating those involved in inflammation [84].

In summary, resveratrol treatment may rebalance oxidative stress and antioxidant competence, preserve mitochondrial morphology by stimulating mitochondrial bioenergetics and biogenesis, reverse the SS mitochondrial damage, and enhance insulin signaling, eventually improving systemic and skeletal muscle insulin resistance. Despite the array of studies demonstrating beneficial effects of resveratrol in metabolic disorders, optimal dose of resveratrol remains elusive in clinical practice, as insufficient effect of resveratrol treatment has been reported in some studies [90]. Therefore, more investigations are necessary to fully understand resveratrol’s therapeutic potential. Additionally, human clinical trials should focus on safe pharmacological doses considering bioavailability and pharmacokinetics of resveratrol.

### Omega-3 Fatty Acids

Omega-3 polyunsaturated fatty acids (PUFA), docosahexaenoic acid (DHA), and eicosatetraenoic acid (EPA) are dietary compounds that are primarily found in fish oil and certain plant oils. Omega-3 PUFAs have been extensively studied for their diverse health benefits including their positive impact on skeletal muscle mitochondria. In several studies, omega-3 fatty acid treatment has been shown to enhance mitochondrial biogenesis and oxidative metabolism. Mice that received 40% fat from fish oil for six weeks of the study period had not only exhibit high metabolic efficiency, but also showed an increased respiratory capacity associated with the increase in gene expression levels of PGC1α/β, and citrate synthase enzyme activity, mitochondrial content [91]. Similarly, a human cell line study also reported that omega-3 treatment significantly induced both glycolytic and oxidative metabolism and increased mitochondrial content [92].

Omega-3 fatty acids have also been implicated in modulating mitochondrial dynamics. In a study by Lionetti et al., 21.8 g of fish oil in 100 g of the diet was given to male rats for six weeks. Compared to high-lard diet group, high-fish oil feeding showed enhanced skeletal muscle mitochondrial fusion, facilitated by mitofusins (MFN1 and MFN2) and OPA1 [93]. Additionally, omega-3 PUFAs also downregulated mitochondrial fission proteins and improved insulin signaling, suggesting that the anti-obesity effects of omega-3 on insulin resistance development may be partly due to changes in mitochondrial dynamic behavior in skeletal muscle [93]. The authors suggested obesity-associated insulin resistance may be highly associated with an increased proinflammatory cytokine level that inhibits mitochondrial fusion, which supports their findings that given the effects of omega-3 PUFA on reducing proinflammatory cytokine levels [93].

### Other Natural Compounds

Other natural compounds known for influencing mitochondrial function have undergone investigation. Berberine, the main active component of Chinese herb *Rhizoma copitidis*, is recognized for its diverse pharmacological actions such as anti-diarrheic, anti-microbial, anti-cancer, anti-inflammatory, anti-arrhythmic, and most recently, hypoglycemic effects [94]. The beneficial effects of berberine on glucose tolerance and insulin sensitivity have shown promise in the treatment of diabetes. While the precise mechanisms of berberine needs further exploration, recent reports show that berberine supplementation protects from high-fat diet-induced mitochondrial dysfunction and hyperglycemia in skeletal muscle, partially attributed to an increase in mitochondrial biogenesis [95]. In an animal study, 5 mg/kg/day berberine treatment for four weeks was found to increase the mitochondrial number and function by promoting AMPK/PGC1α signaling pathway [94], which was corroborate in an isolated skeletal muscle of both fast- and slow-type muscle fibers [96]. Notably, the activation of AMPK by berberine may be dependent on SIRT1, as SIRT1-knockdown cells blocked AMPK activation and prevented the effect of berberine on diet-induced insulin resistance [95].

Several flavonoids have also demonstrated similar effects on skeletal muscle mitochondria. Flavonoids from mulberry leaf, for example, improved mitochondrial function in L6 muscle cells via AMPK/PGC1α signaling pathway and improved insulin resistance [97]. Similarly, dietary quercetin supplementation was reported to decrease high-fat diet-induced fat accumulation and insulin resistance [98]. In this study, 17 mg/kg of quercetin aglycone treatment for nine weeks increased skeletal muscle mitochondria number and decreases incomplete β-oxidation, establishing mitochondrial function comparable to that of lean counterparts [98]. Similarly, tangeretin, a flavonoid from the peels of citrus fruits, increases mitochondrial content and activates mitochondrial biogenesis signaling axis via AMPK/PGC1α signaling pathway [99].

In summary, dietary bioactive compounds such as polyphenolic compound resveratrol, omega-3 fatty acids, berberine, and several flavonoids have been investigated in mitochondrial research. The results implicate an association between the effects of these phytonutrients on mitochondrial and skeletal muscle health. Based on these findings, these bioactive compounds exert their effects through SIRT1/AMPK/PGC1α signaling pathways. As with many dietary bioactive compounds, future investigations should focus on developing clinical implications and determining realistic clinical doses.

## Conclusions and Future Perspectives

Mitochondria, a cornerstone of cellular health, plays a vital role in skeletal muscle well-being. Disruptions in mitochondrial homeostasis, exhibiting as dysfunctional mitochondria and reduced mitochondrial content, pose profound threats to skeletal muscle health. The intricate balance of fusion and fission cycles, coupled with tightly regulated biogenesis and mitophagy, constitutes the machinery coordinating mitochondrial quality control. In obese muscles, intramuscular lipid accumulation disrupts these machineries, causing perturbations in mitochondrial signaling cascades, particularly involving the SIRT1/AMPK/PGC1α pathways. This review explores various interventions aimed at mitigating mitochondrial dysfunction, encompassing strategies including caloric restriction, resveratrol, omega-3 fatty acids, and other natural compounds. While these interventions show promise in in vivo and ex vivo studies, the precise molecular associations between lifestyle modifications, nutraceuticals, and obesity-induced mitochondrial dysfunction remain elusive. Therefore, it is imperative to bridge this gap for a comprehensive understanding. Robust research efforts will provide a foundation for targeted strategies to mitigate obesity-induced mitochondrial dysfunction and preserve skeletal muscle health.

## Data Availability

No datasets were generated or analysed during the current study.

## Abbreviations

WHO: World Health Organization
BMI: Body mass index
T2DM: Type 2 diabetes mellitus
ATP: Adenosine triphosphate
MHC: Myosin heavy chain
OXPHOS: Oxidative phosphorylation
mtDNA: Mitochondrial DNA
OMM: Outer mitochondrial membrane
IMM: Inner mitochondrial membrane
ETC: Electron transport chain
SS: Subsarcolemmal
IMF: Intermyofibrillar
ROS: Reactive oxygen species
PINK1: PTEN-induced putative kinase 1
SQSTM1: Sequestome-1
NBR1: BRCA1 gene 1 protein
NDP52: Nuclear dot protein 52
OPTN: Optineurin
LC3: Microtubule-associated 1A/B-light chain 3
PGC1α: Peroxisome proliferator-activated receptor γ coactivator 1α
SIRT1: Silent information regulator 2 homologue 1
MAPK: Mitogen-activated protein kinase
LKB1: Liver kinase B1
AMPK: Adenosine monophosphate-activated protein kinase
NAD: Nicotinamide adenine dinucleotide
NRF: Nuclear respiratory factor
TFAM: Mitochondrial transcription factor A
MFN1/2: Mitofusin 1/2
OPA1: Optic atrophy 1
DRP1: Dynamin-related protein 1
ER: Endoplasmic reticulum
FIS1: Fission protein 1
PUFA: Polyunsaturated fatty acids
DHA: Docosahexaenoic acid
EPA: Eicosatetraenoic acid
